# Cognitive impairment in patients with Fibromyalgia syndrome as assessed by the Mini-Mental State Examination

**DOI:** 10.1186/1471-2474-10-162

**Published:** 2009-12-21

**Authors:** Jose Rodríguez-Andreu, Rosario Ibáñez-Bosch, Amparo Portero-Vázquez, Xavier Masramon, Javier Rejas, Rafael Gálvez

**Affiliations:** 1 Department of Rheumatology, Hospital Virgen de la Victoria, Málaga, Spain; 2 Department of Rheumatology, Hospital de Navarra, Pamplona, Spain; 3 Department of Rehabilitation, Hospital Xeral-CALDE, Lugo, Spain; 4 European Biometric Institute, Barcelona, Spain; 5 Health Outcomes Research Department, Medical Unit, Pfizer España, Alcobendas, Spain; 6 Pain and Palliative Care Unit, University Hospital Virgen de las Nieves, Granada, Spain

## Abstract

**Background:**

This study evaluated the frequency of cognitive impairment in patients with Fibromyalgia syndrome (FMS) using the Mini Mental State Examination (MMSE).

**Methods:**

We analyzed baseline data from all 46 patients with FMS and 92 age- and sex-matched controls per diagnosis of neuropathic (NeP) or mixed pain (MP) selected from a larger prospective study.

**Results:**

FMS had a slight but statistically significant lower score in the adjusted MMSE score (26.9; 95% CI 26.7-27.1) than either NeP (27.3; 95% CI 27.2-27.4) or MP (27.3; 27.2-27.5). The percentage of patients with congnitive impairment (adjusted MMSE ≤ 26) was numerically higher in FMS (15%; 95% CI 6.3-29) compared with NeP (5%; 95% CI 1.8-12.2) or MP (5%; 95% CI 1.8-12.2) and higher than in the same age stratum of the general population (0.05%).

**Conclusions:**

Compared with the population reference value, patients with FMS showed high frequency of cognitive impairment.

## Background

Fibromyalgia syndrome (FMS) is a common chronic musculoskeletal disorder characterized by the presence of widespread pain and multiple tender points on physical examination [1]. Other important accompanying symptoms of FMS are fatigue, sleep disturbance, psychological distress and cognitive disturbance [1]. In 1990, the American College of Rheumatology (ACR) published diagnostic criteria for FMS -namely, widespread pain (both sides of the body, above and below the waist, and in the cervical spine, anterior chest, thoracic spine or low back), and pain upon digital palpation in at least 11 of 18 specified tender point sites [2], although it was not officially recognized as an illness by the World Health Organization until 1992. [3]

FMS is a widespread disorder of unknown etiology that affects an estimated 1-4% of the general population. [4] It may occurs in 2.1 -5.7% of the general adult population, making up 10 -20% of rheumatologic consultations and 5 -8% of primary care consultations, being the most frequent cause of general and chronic skeletal muscular pain. [5-7] Women are about nine times more likely to develop FMS than men. [4,7] The symptoms of FMS can be prolonged and debilitating. It negatively affects the lives of patients, the people around them and the environment in which they live. It is one of the rheumatic illnesses with the greatest impact on patient quality of life, having negative consequences on physical capability, intellectual activity, emotional condition, personal relationships, professional career and mental health, to the extent where the patient requires multiple intervention strategies. [8,9] In recent years, FM has acquired greater significance and has become a first-order public health problem due to prevalence, insufficient knowledge of its cause and the mechanisms that produce it, and dissatisfaction of patients and professionals with current therapeutic approaches.

Over 50% of the patients with FMS complain of memory decline and mental confusion [10]. Cognitive problems, such as forgetfulness and concentration difficulties, are among the more severe symptoms of patients with this condition [11]. Results from neuropsychological tests show that patients with FMS have impairments in working, episodic and semantic memory, as well as in selective attention [12]. Despite the importance of the cognitive symptoms and the experts' recommendation to evaluate them as part of the standard FMS assessment,[5] information on the frequency of cognitive problems is limited to a few studies as reviewed by Glass [12]. These studies used either subjective complaints or the more complicated and less clinically feasible of neuropsychological test evaluations.

The objective of this post-hoc exploratory analysis was to evaluate the frequency of cognitive impairment in patients with FMS using a brief and widely used quantitative measure of the cognitive status in adults such as the Mini Mental State Examination.

## Methods

### Study population and overall design

The original sample was comprised of 1,519 patients of both gender, aged ≥ 18 years, enrolled into an open label, pragmatic, prospective, multicenter study conducted in specialized pain care clinics (pain clinics, departments of neurology, rheumatology clinics and department of rehabilitation) throughout Spain between 2002 and 2003 [13]. This study assessed the effectiveness of gabapentin for neuropathic pain (NeP) and mixed pain (MP) of broad origin. The eligibility criteria included: patients of both gender aged ≥ 18 years with a clinical diagnosis of neuropathic or mixed pain. Patients with known hypersensitivity to gabapentin or its ingredients, pregnant or nursing women, and patients receiving other analgesic drugs indicated for neuropathic pain were excluded from the evaluation. Patients were consecutively enrolled by non-probabilistic sampling. Geographic representativeness was achieved by an unbiased selection of the specialized care centers throughout the whole country, and a random enrollment of investigators in each selected center. Thus, centre participation was proportional to the density of the Spanish population.

From the original sample, we selected for this analysis of the baseline data all patients with a diagnosis of FMS according to ACR criteria as well as two age (± 1 year) and sex-matched controls per diagnosis of NeP or MP.

The study source of data was conducted in compliance with Spanish legal regulations for observational, post-marketing studies of drugs. The study was reviewed and approved by a Clinical Research Ethics Committee and conducted in compliance with the Declaration of Helsinki. A written informed consent was obtained from patients before study entry.

### Description of scales

Cognitive function was measured by administering the Spanish version of the Mini-Mental State Examination (MMSE), which has been validated and adjusted by age and schooling time [14]. In this study, a score of 26 or less was considered as any degree of cognitive impairment (CI)[14]. Pain was assessed with the short form of the McGill Pain Questionnaire (SF-MPQ) [15] Symptoms of anxiety and depression were assessed by means of the Covi [16] and Raskin [17] scales, respectively. These scales were used in the analysis for possible confounding factors adjustment.

### Statistical methods

We conducted a post-hoc statistical analysis of data. Crude scores of the MMSE were corrected by age and years of schooling [14]. A descriptive analysis was performed, including central tendency and dispersion statistics, or ratios for categorical variables, and normal distribution testing using the Kolmogorov-Smirnov test. A homogeneity analysis among three different patient groups was also performed. This analysis used parametric and robust (Welch and Brown-Forsythe robust tests) analysis of variance (ANOVA), depending on the variable type and distribution. Categorical variables were compared using a Chi-square test with the Yates correction, and the Fisher's exact test in case of small sample sizes. The linear Chi-square test was used to compare ordered ordinal variables. All tests were two-sided, and an error of α < 0.05 was accepted as significant.

Crude scoring in MMSE which already is corrected by age and schooling time was adjusted for confounders by means of a multivariate lineal regression model. Confounders included in the model were symptoms of anxiety and depression, last week average intensity of pain, sex and previous exposition (before entering the study) to any analgesic drugs or treatment for pain (i.e.: antiepileptics, tricyclics, NSAIDs, etc.). Adjusted scoring was then used to classify subjects as having CI (adjusted MMSE ≤ 26) and their 95% confidence intervals calculated from the values predicted by the regression analysis. The adjusted CI prevalence was compared in the different subgroups assessed by a Chi^2 ^test and adjusted scoring by ANOVA.

SPSS version 17.0 for Windows was used as the Statistical package.

## Results

Patients included in our analysis were mostly middle-aged women who were less than two years after the diagnosis of their condition. Although there were no statistically significant differences, less than one third of FMS patients had received previous analgesic treatment as compared with two thirds of patients with NeP or MP. Patients in the three study groups had moderate to severe pain as judged by the pain score in the VAS of the SF-MPQ. Patients with FMS exhibited significantly higher levels of anxiety and depressive symptoms compared with the other two study groups (Table 1).

**Table 1:** Demographic characteristics and baseline scoring in the short-form McGill Pain Questionnaire of patients with neuropathic pain or neuropathic and nociceptive mixed pain observed in the study.

Variable	FMS (n = 46)	NeP (n = 92)	MP (n = 92)	p
Age (years)	50.8 (47.8-53.8)	50.9 (48.8-53.0)	50.7 (48.6-52.8)	0.989 (F = 0.01; df = 229)
Sex, female (%)	85 [71 - 94]	85 [76 - 91]	85 [76 - 91]	1.000 (X^2 ^= 0.002; df = 2)
Time since diagnosis (years)	1.8 (1.3-2.3)	1.4 (0.7-2.1)	1.9 (1.2-2.6)	0.434 (F = 0.69; df = 229)
0 - 12 months (%)	52 [37 - 67]	75 [65 - 83]	61 [50 - 71]	0
12 - 24 months (%)	15 [6 - 29]	10 [5 - 18]	11 [5 - 19]	0
> 24 months (%)	33 [20 - 48]	15 [9 - 24]	28 [19 - 39]	0
Previous analgesic treatment (%)	30 [187-46]	65 [59-79]	67 [57-77]	0.001 (X^2 ^= 19.68; df = 2)

Pain score (McGill)				
VAS (0 to 100):	70.8 (66.2-75.4)	71.7 (67.5-75.9)	72.6 (69.1-70.1)	0.849 (F = 0.16; df = 229)
PPI (0 to 5):	2.6 (2.3-2.9)	2.7 (2.5-3.0)	2.9 (2.7-3.0)	0.294 (F = 1.23; df = 229)
Self-perceived symptoms of;				
Anxiety (COVI; 0-15)	7.0 (6.2-7.9)	5.8 (5.2-6.4)	5.8 (5.2-6.3)	0.028 (F = 3.65; df = 229)
Depression (RASKIN; 0-15)	8.4 (7.4-9.4)	6.4 (5.7-7.1)	6.5 (5.9-7.1)	0.001 (F = 7.39; df = 229)

FMS = Fibromyalgia Syndrome; NeP = Neurophatic Pain; MP = Mixed Pain; VAS = Visual Analogue Scale; PPI = Present Pain Intensity. Values are given as observed frequency, proportion, or mean (standard deviation) [95% confidence interval]. Except where noted, all differences are non-significant. F = F statistic (ANOVA); df = degree of freedom. X^2 ^= Chi^2 ^test.

**Figure 1:**
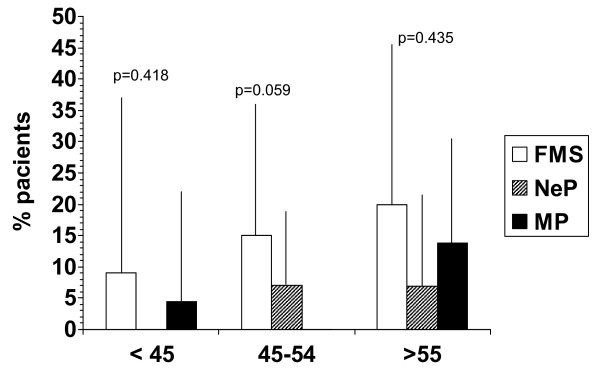
**Frequency of cognitive impairment according to age and study group**. FMS = Fibromyalgia Syndrome; NeP = Neurophatic Pain; MP = Mixed Pain. Bars are mean percentage with upper limit 95% CI.

Patients with FMS had a slight but statistically significant lower score in the adjusted MMSE score (mean 26.9; 95% CI: 26.7-27.1) than either patients with NeP (27.3; 27.2-27.4) or MP (27.3; 27.2-27.5). In fact, although there were no statistically significant differences among the study groups in the proportion of patients with congnitive impairment (i.e. MMSE <26), this frequency was numerically higher in patients with FMS (15%; 95% CI 6.3-29) than in patients with NeP (5%; 95% CI 1.8-12.2) or MP (5%; 95% CI 1.8-12.2). In the univariate analysis, patients with at least moderate symptoms of anxiety or depression (that is, those patients with scores equal or greater than 9 in the COVI or RASKIN, respectively) had higher rates of cognitive impairment. However, these rates were not significantly different among study groups (Table 2). Similarly, rates of cognitive impairment did not vary according to pain severity in any of the groups (Table 2). As expected, the frequency of cognitive impairment appeared to increase with age, particularly in FMS group (Figure 1). There were no significant differences among study groups in the rates of cognitive impaiment in each age strata. However, a trend towards significance was found in the group of patients 45 to 54 years old (Figure 1).

**Table 2:** Cognitive deficit in Fibromyalgia patients compared with subjects with neuropathic pain or mixed pain. Comparisons are overall, by level of symptoms of anxiety and depression, and by severity of present pain.

	**FMS** **(n = 46)**	**NeP** **(n = 92)**	**MP** **(n = 92)**	**P**
**MMSE scoring** (Range 0-30); mean (95% CI)	26.9 (26.7-27.1)	27.3 (27.2-27.4)	27.3 (27.2-27.5)	0.001 (F = 7.32; df = 229)
**% patients with cognitive deficit** (MMSE < 26); n, (%) [95% CI]	7 (15%) [6.3-29]	5 (5%) [1.8-12.2]	5 (5%) [1.8-12.2]	0.076 (X^2 ^= 5.146; df = 2)
**% patients with cognitive deficit by** **Presence of symptoms of**				
Anxiety (COVI ≥ 9)	7/14 (50%) [23-77]	5/17 (29%) [10.3-56]	5/15 (33%) [11.8-61.6]	0.467 (X^2 ^= 1.522; df = 2)
Depression (RASKIN ≥ 9)	7/19 (37%) [16.3-61.6]	5/20 (25%) [8.6-49.1]	5/21 (24%) [8.2-47.2]	0.607 (X^2 ^= 0.999; df = 2)
**Intensity of pain (average last week)**				
Mild (< 40)	1/2 (50%) [12.6-98.7]	1/7 (14%) [0.4-57.9]	0-3 (0%) [0-70.8]	0.328 (X^2 ^= 2.229; df = 2)
Moderate (≥40<70)	2/15 (13%) [1.6-40.5]	2/26 (8%) [0.9-25.1]	1/26 (4%) [0-19.6]	0.537 (X^2 ^= 1.243; df = 2)
Severe (≥ 70)	4/29 (14%) [3.9-31.7]	2/59 (3%) [0.4-11.7]	4/63 (6%) [1.8-15.5]	0.177 (X^2 ^= 3.464; df = 2)

FMS = Fibromyalgia Syndrome; NeP = Neurophatic Pain; MP = Mixed Pain; MMSE scoring adjusted by age, sex and schooling. Cognitive deficit: MMSE ≤ 26 pts after adjusting by age, sex, schooling, present intensity of pain (PPI item) and severity of symptoms of anxiety (COVI) and depression (RASKIN). CI = Confidence interval; F = F statistic (ANOVA); df = degree of freedom. X^2 ^= Chi^2 ^test.

## Discussion

Our results on this post-hoc analysis showed that patients with FMS were associated with a high frequency of cognitive impairment (15%, 95% CI 6.3-29), which was substantially above the known prevalence of cognitive impairment reported for the Spanish general population within the same age stratum (i.e. 0.05%)[18]. Rates of cognitive impairment were not different among the study groups according to age, pain severity or the presence of anxiety or depressive symptoms. Noteworthy, however, is that regardless of the diagnosis, all patients with cognitive impairment also exhibited significant symptoms of anxiety and depression (i.e. a score equal or greater than 9 in the COVI and RASKIN scales, respectively). Despite our cross-sectional design, this finding strongly suggests that the cognitive impairment we observed in all these painful conditions may be associated with underlying psychological symptoms. Results from previous studies support this view. Although Park et al did not find a correlation between the impairment of cognitive performance and depressive or anxiety symptoms in patients with FMS [19], other authors have found an association between cognitive impairment and anxiety symptoms [20] or depressive symptoms [21,22]. Landrø et al found that only the subgroup of patients with FMS and a lifetime history of major depression showed memory impairment when compared with healthy controls [21]. Suhr showed that depression was significantly related to memory performance in patients with FMS or other pain chronic disorders such as chronic headache or osteoarthritis [22]. Whether the cognitive impairment in FMS patients is more severe or frequent than in patients with neuropathic pain or mixed pain requires still more research as numerically differences observed in this study did not reach statistical significance due to the small sample of the study.

Our analysis has several limitations. Besides its small sample size, only a low proportion of patients with FMS (3%) were among the patients originally recruited. This may be due the type of participating specialist and/or because consider this condition as a form of neuropathic pain is still controversial. Another possible limitation is the heterogeneous nature of this condition; suggesting that patients with FMS from our study could not be well classified to some extent.

## Conclusion

To conclude, patients with FMS showed a high frequency of cognitive impairment, particularly when compared with known prevalence of this condition in the general population. This cognitive impairment remained higher after adjustment by the presence of anxiety and depressive symptoms. Further studies with larger samples of patients are required to confirm our results, to support possible differences in frequency as compared with other painful conditions than FMS, as well as to evaluate the potential impact of this mild impairment of the cognitive function on the patient's daily functionality and/or on the risk of progression to a more severe deterioration.

## Abbreviations

FMS: Fibromyalgia syndrome, NeP: Neuropathic pain, MP: Mixed pain, MMSE: Mini-Mental State Examination, CI: Cognitive impairment, SF-MPQ: Short Form of the McGill Pain Questionnaire, ANOVA: Analysis of variance.

## Competing interests

The authors of this manuscript state that all of the authors have seen and agreed to the submitted version of the manuscript and with the listing of the authors. All authors have contributed substantially in the manuscript preparation, interpretation of results or study design. The principal authors takes full responsibility for the data presented in this study, analysis of the data, conclusions, and conduct of the research, and had full access to those data and has maintained the right to publish any and all data independent of any third party.

The study was funded by an unrestricted grant from Pfizer España. Javier Rejas is employed of Pfizer España. Xavier Masramón is employed by European Biometric Institute, and independent company contracted for Pfizer España to collaborate in data analysis and logistic of the study.

## Authors' contributions

JRA, RIB, and APV participated in the design of this study, interpretation of data and the writing of this manuscript. RG participated in the preparation of the manuscript, interpretation of data and in the literature review and extraction. XM and JR participated in the analysis of data and in the preparation of the manuscript. All of the authors read and approved the content of the manuscript.

## Pre-publication history

The pre-publication history for this paper can be accessed here:

http://www.biomedcentral.com/1471-2474/10/162/prepub

## References

[B1] Mease P Fibromyalgia syndrome: review of clinical presentation, pathogenesis, outcome measures, and treatment J Rheumatol Suppl 2005 75 6 21 16078356

[B2] Wolfe F Smythe HA Yunus MB Bennett RM Bombardier C Goldenberg DL Tugwell P Campbell SM Abeles M Clark P The American College of Rheumatology 1990 criteria for the classification of fibromyalgia Arthritis Rheum 1990 33 160 72 10.1002/art.1780330203 2306288

[B3] Consensus Document on Fibromyalgia. The Copenhagen Declaration Journal of Musculoskeletal Pain 1993 1 New York: Haworth Press

[B4] Wolfe F Ross K Anderson J Russell IJ Hebert L The prevalence and characteristics of fibromyalgia in the general population Arthritis Rheum 1995 38 19 28 10.1002/art.1780380104 7818567

[B5] Carmona L Ballina FJ Gabriel R Laffon A EPISER Study Group: The burden of musculoskeletal diseases in the general population of Spain: results from a nation-wide study Ann Rheum Dis 2001 60 1040 5 10.1136/ard.60.11.1040 11602475 PMC1753418

[B6] Fundación Grünenthal, Sociedad Española de Reumatología Estudio EPIDOR: estudio epidemiológico del dolor en España 2003 Madrid: Edipharma

[B7] Ruiz I Ubago MC Bermejo MJ Plazaola J Olry A Hernández E Differences in, sociodemographic clinical, psychosocial and health care characteristics between men and women diagnosed with fibromyalgia Rev Clin Esp 2007 207 433 439 10.1157/13109832 17915163

[B8] Tornero J Vidal J Impacto social y económico de las enfermedades reumáticas: la discapacidad laboral Rev Esp Reumatol 1999 26 347 366

[B9] Buckhardt CS Goldenberg D Crofford L Gerwin R Gowens S Jackson K Kugel P McCarberg W Rudin N Schanberg L Taylor AG Taylor J Turk D Guideline for the management of fibromyalgia syndrome pain in adults and children 2005 Glenview (IL): American Pain Society (APS) 109 (Clinical practice guideline; no. 4)

[B10] Katz RS Heard AR Mills M Leavitt F The Prevalence and Clinical Impact of Reported Cognitive Difficulties (Fibrofog) in Patients With Rheumatic Disease With and Without Fibromyalgia J Clin Rheumatol 2004 10 53 58 10.1097/01.rhu.0000120895.20623.9f 17043464

[B11] Bennett RM Jones J Turk DC Russell IJ Matallana L An internet survey of 2,596 people with fibromyalgia BMC Musculoskelet Disord 2007 8 27 10.1186/1471-2474-8-27 17349056 PMC1829161

[B12] Glass JM Fibromyalgia and cognition J Clin Psychiatry 2008 69 Suppl 2 20 4 18537459

[B13] Povedano M Gascón J Gálvez R Ruiz M Rejas J Cognitive function impairment in patients with neuropathic pain under standard conditions of care J Pain Symptom Manage 2007 33 78 89 10.1016/j.jpainsymman.2006.07.012 17196909

[B14] Blesa R Pujol M Aguilar M Santacruz P Bertran-Serra I Hernández G Sol JM Peña-Casanova J Clinical validity of the "mini-mental state" for Spanish speaking communities Neuropsychologia 2001 39 NORMACODEM Group 1150 1157 10.1016/S0028-3932(01)00055-0 11527552

[B15] Melzack R The short-form McGill Pain Questionnaire Pain 1987 30 191 197 10.1016/0304-3959(87)91074-8 3670870

[B16] Lipman RS Differentiating anxiety and depression in anxiety disorders: use of rating scales Psychopharmacol Bull 1982 18 69 77 7156301

[B17] Raskin A Schulterbrandt J Reating N McKeon JJ Replication of factors of psychopathology in interview, ward behaviour and self report ratings of hospitalized depressives J Nerv Ment Dis 1969 148 87 98 5768895 10.1097/00005053-196901000-00010

[B18] Bermejo-Pareja F Benito-León J Vega S Medrano MJ Román GC Neurological Disorders in Central Spain (NEDICES) Study Group J Neurol Sci 2008 264 1-2 63 72 10.1016/j.jns.2007.07.021 17727890

[B19] Park DC Glass JM Minear M Crofford LJ Cognitive function in fibromyalgia patients Arthritis Rheum 2001 44 2125 2133 10.1002/1529-0131(200109)44:9<2125::AID-ART365>3.0.CO;2-1 11592377

[B20] Grace GM Nielson WR Hopkins M Berg MA Concentration and memory deficits in patients with fibromyalgia syndrome J Clin Exp Neuropsychol 1999 21 477 487 10.1076/jcen.21.4.477.876 10550807

[B21] Landrø NI Stiles TC Sletvold H Memory functioning in patients with primary fibromyalgia and major depression and healthy controls J Psychosom Res 1997 42 297 306 10.1016/S0022-3999(96)00301-7 9130186

[B22] Suhr JA Neuropsychological impairment in fibromyalgia: relation to depression, fatigue, and pain J Psychosom Res 2003 55 321 329 10.1016/S0022-3999(02)00628-1 14507543

